# Comparison of Application Effects of Different Hemostasis Methods After Ischemic Cerebrovascular Intervention

**DOI:** 10.3389/fsurg.2022.850139

**Published:** 2022-03-07

**Authors:** Yanli Zhou, Chenghua Xu

**Affiliations:** Department of Neurology, Taizhou First People's Hospital, Taizhou, China

**Keywords:** ischemic cerebrovascular diseases, cerebrovascular intervention, hemostatic methods, artery compressor, vascular closure devices

## Abstract

**Objective:**

To explore the effects of two different hemostasis methods, namely, arterial compression devices and vascular closure devices, in the ischemic cerebrovascular intervention to provide a theoretical basis for clinical selection of hemostasis methods.

**Methods:**

A total of 302 patients who underwent ischemic cerebrovascular intervention in our hospital from January 2016 to December 2020 were selected as the research subjects and randomly divided into the control group (*n* = 151) and the observation group (*n* = 151). The patients in both groups underwent cerebrovascular intervention. The patients in the control group were treated with an artery compressor for hemostasis after the operation, while those in the observation group were treated with vascular closure devices for hemostasis. The hemostatic indexes and vascular parameters at the puncture site before and after the operation were compared between the two groups. The comfort level of the patients was assessed at 6, 12, and 24 h after the operation with the Kolcaba Comfort Scale score, and the postoperative complications were recorded.

**Results:**

There was no significant difference in the success rate of hemostasis between the two groups (*p* > 0.05). The hemostatic time and immobilization time of (2.69 ± 0.62) min and (4.82 ± 0.93) h in the observation group were lower than those in the control group with (16.24 ± 3.58) min and (7.94 ± 1.86) h (*p* < 0.05). The differences in the minimum inner diameter of the puncture site and its nearby vessels and the peak velocity of blood flow between the two groups before and after the operation were not statistically significant within or between groups (*p* > 0.05). The scores of the Kolcaba comfort scale in the observation group (80.16 ± 8.49) and (93.65 ± 9.26) at 6 and 12 h, respectively, after the operation, were higher than those in the control groups (72.08 ± 7.54) and (85.49 ± 8.63) (*p* < 0.05). The 24 h postoperative Kolcaba comfort scale score was (97.54 ± 9.86) in the observation group and (96.82 ± 9.64) in the control group, and the difference was not statistically significant (*p* > 0.05). In the control group, there were 7 cases of dysuria, 12 cases of low back pain, 14 cases of sleep disorder, 20 cases of mental stress, and 5 cases of wound bleeding, and the total incidence of complications was 38.41% (58/151). In the observation group, there were 4 cases of dysuria, 8 cases of low back pain, 10 cases of sleep disorder, 14 cases of mental stress, and 3 cases of wound bleeding, and the total incidence of complications was 25.83% (39/151). The total incidence of complications in the observation group was lower than that in the control group (*p* < 0.05).

**Conclusion:**

For patients with ischemic cerebrovascular disease undergoing femoral artery puncture intervention, the use of vascular closure devices can stop the bleeding quickly, which can significantly shorten the bleeding time, and the postoperative braking time of patients is short, with high comfort and fewer complications.

## Introduction

Cerebrovascular disease refers to acute and chronic cerebrovascular diseases caused by various reasons. As a common and frequently occurring disease in the nervous system, cerebrovascular disease is currently one of the three major diseases leading to human death, second only to cardiovascular disease and cancer (1, 2). Ischemic cerebrovascular diseases mainly include transient ischemic attack and cerebral infarction, accounting for about 80% of all strokes, and the proportion is higher in elderly patients. At present, the clinical treatments of ischemic cerebrovascular diseases mainly include medication, surgery, and intervention (3, 4). Traditional medication has little effect on the formed plaques, especially the moderate and severe vascular stenosis. Carotid endarterectomy was once the gold standard for the treatment of carotid stenosis, but it has been limited due to its large surgical trauma and a relatively small scope of indications (5, 6). Interventional therapy belongs to minimally invasive surgery, which is usually performed under local anesthesia. Due to its advantages of not damaging brain nerve fibers, a short time of blocking the blood flow during surgery, and its applicability to high-level stenosis, it is one of the most important methods for the treatment of cerebral artery stenosis after the surgical carotid endarterectomy (7, 8). To avoid vasospasm during cerebral vascular intervention, the femoral artery, and the inguinal artery are usually used as puncture vessels in clinics. However, coagulation disorders may occur due to the treatment with anticoagulant and antiplatelet drugs during the operation, resulting in massive and continuous bleeding, which affects the prognosis of patients (9, 10). Therefore, it is extremely important to select a hemostatic method with few complications, high comfort, and easy caring. Both the arterial compression devices and the vascular staplers are new hemostatic devices. Studies have shown that, compared with traditional artificial compression hemostasis, the hemostatic effect is better (11, 12). The purpose of this study was to investigate the application effects of two different hemostasis methods, namely, the artery compressor and the arterial suture device in the ischemic cerebrovascular intervention, to provide a theoretical basis for clinical selection of hemostasis methods.

## Materials and Methods

### Patients

A total of 302 patients, who underwent ischemic cerebrovascular intervention in our hospital from January 2016 to December 2020, were selected as the research subjects. There were 177 men and 125 women; the age ranged from 40 to 78 years, with an average age of (55.86 ± 9.82) years; the modified Rankin scale (mRS) was (3.15 ± 0.55) points. Inclusion criteria: preoperatively enhanced CT, MRI, and digital subtraction angiography were performed in all patients to identify the stenotic cerebral artery vessels and sites; all the patients underwent cerebrovascular intervention—those who are conscious before surgery; Preoperative examination of coagulation function, liver and kidney function, and heart function is normal. Exclusion criteria: the patients with systemic infectious diseases, the patients with urinary system diseases, the patients with back pain, the patients with severe respiratory diseases, and the patients with mental and cognitive dysfunction. All the patients were randomly divided into the control group (*n* = 151) and the observation group (*n* = 151). There was no significant difference in general data between the two groups (*p* > 0.05), as shown in Table 1.

**Table 1:** Comparison of general data between the two groups.

**Group**	**Gender**		**Age (years)**	**mRS score (points)**
	**Male**	**Female**		
Control group (*n* = 151)	92	59	55.67 ± 9.68	3.12 ± 0.56
Observation group (*n* = 151)	85	66	56.05 ± 9.95	3.18 ± 0.54
t/χ^2^	0.669		0.336	0.948
*P*	0.414		0.737	0.344

### Treatment Methods

All the patients in the two groups underwent cerebrovascular intervention; before the operation, all the patients had undergone routine electrocardiogram (ECG) and routine blood tests, as well as liver and kidney functions and bleeding and coagulation functions. Aspirin 300 mg/d and clopidogrel 75 mg/d were routinely administered orally to inhibit platelet aggregation for 3 days before surgery. Stent placement: The femoral approach was adopted, and a 6F arterial sheath was inserted into the vertebral artery, while an 8F arterial sheath was inserted into the internal carotid artery for systemic heparinization. The site and the degree of arterial stenosis and collateral circulation in the ischemic area were determined by angiography. Under the guidance of the wire and the path diagram, a 6F/8F guide catheter was inserted into the proximal end of the lesion. The microwire is inserted along that guide catheter, and the head end of the microwire is inserted through the vascular stenosis and is positioned at the distal end thereof; then, the balloon dilatation catheter was inserted along the microwire to expand the stenosis, and the stent was finally placed at the stenosis. Distal brain protection devices are used for patients with internal carotid artery stenosis. Angiography was performed to observe the dilation of the stenosis, and balloon dilatation was performed if necessary. The intra-cervical and vertebral artery angiography and intracranial angiography were repeated. The arterial dilation after stent release and the blood supply to each branch of the intracranial segment was observed. If no abnormality was found, the operation was terminated.

The control group was treated with arterial compression hemostasis (Model: YM-GU-1229, Manufacturer: Tianjin Yimei Company) after the operation. The arterial compression hemostat was mainly composed of an elliptical pressure plate, a fixed adhesive tape, a spiral handle, a base, and a dial. The operator first confirmed the position of the femoral artery puncture point, applied pressure to the puncture point, pulled out the artery sheath, covered the puncture point with gauze, and then pressed the artery puncture point with an oval pressure plate, and tightly wound the femoral part with the fixed tape. Then, the handle of the screw was rotated clockwise for 6–8 weeks to pressurize the puncture point to confirm that the puncture point could bear the pressure of the pressure plate and to avoid the puncture point bleeding due to excessive pressure. They began to relax after dressing for 2–3 h and rotated the handle counterclockwise for 1 turn. About 2–3 h later, the handle was rotated counterclockwise for 1 turn, and then, it was relaxed every 30–40 min for 1 turn. About 2 h later, the patient could slowly move the lower limb, and, about 6 h later, the patient could slowly turn over, observe exudation around the puncture point, observe the swelling of the operative limb, completely relax about 8 h later, and continue to observe for 30 min. If there is no bleeding, the hemostatic presser can be removed, and the patient moves slightly on the bed.

Hemostasis in the observation group was performed using the vascular closure devices (Model: Perclose Pro Glide, Manufacturer: Abbo TT Labora Tories); after the vascular condition was evaluated, the Perclose Pro Glide suture device was used for the local suture of the punctured vessels, and 0.035 inch wire was replaced through the catheter sheath to enter the femoral artery, followed by the guidewire into the vascular suture device, and then out of the guidewire, and push the suturing device until there is an obvious pulse blood flow in the labeled tube. Position the device at about 45°, raise the handle to expand the pin, and press the piston assembly to complete the endovascular knot when there is resistance in the pull-back. After exiting the vascular suture instrument, the suture modifier was used to push the suture knot onto the femoral artery wall, and finally, the suture knot was tightened, and the excess suture part was cut to complete the operation.

### Observation Indicators

(1) Hemostatic effect indicators: The success rate of hemostasis, postoperative hemostasis time, and braking time of the patients in the two groups were recorded. Among them, the criteria for successful hemostasis were as follows: After a one-time suture or compression, there was no bleeding at the puncture point, and the bleeding did not occur again at the puncture point until discharged. Hemostasis time: the time from the removal of the arterial sheath to the completion of hemostasis. Braking time: the time from hemostasis to getting out of bed.

(2) Vascular parameters of the puncture site: The minimum inner diameter of the femoral artery within 3 cm of the puncture site and the nearby area, the peak systolic blood flow velocity, and the formation of femoral artery thrombosis were detected preoperatively, and 3 days after the operation, respectively.

(3) The comfort level during hospitalization: Kolcaba comfort scale (13) was used to score the patients at 6, 12, and 24 h after hemostasis. The scale includes physiological dimension, psycho-spiritual dimension, socio-cultural dimension, environmental dimension, and the overall comfort level. The full score is 120, and the higher the score, the higher the patient's comfort level.

(4) Complications: The occurrence of complications such as dysuria, low back pain, sleep disorder, mental stress, and wound bleeding were recorded after the operation.

### Statistical Methods

All data were processed with SPSS 22.0 statistical software. The enumeration data were examined by the X^2^ test and expressed by [*n* (%)]; the measurement data were examined by *t*-test and expressed by (**x** ± s). The difference is statistically significant when *p* < 0.05.

## Results

### Comparison of Hemostatic Efficacy Indicators Between the Two Groups

**Figure 1:**
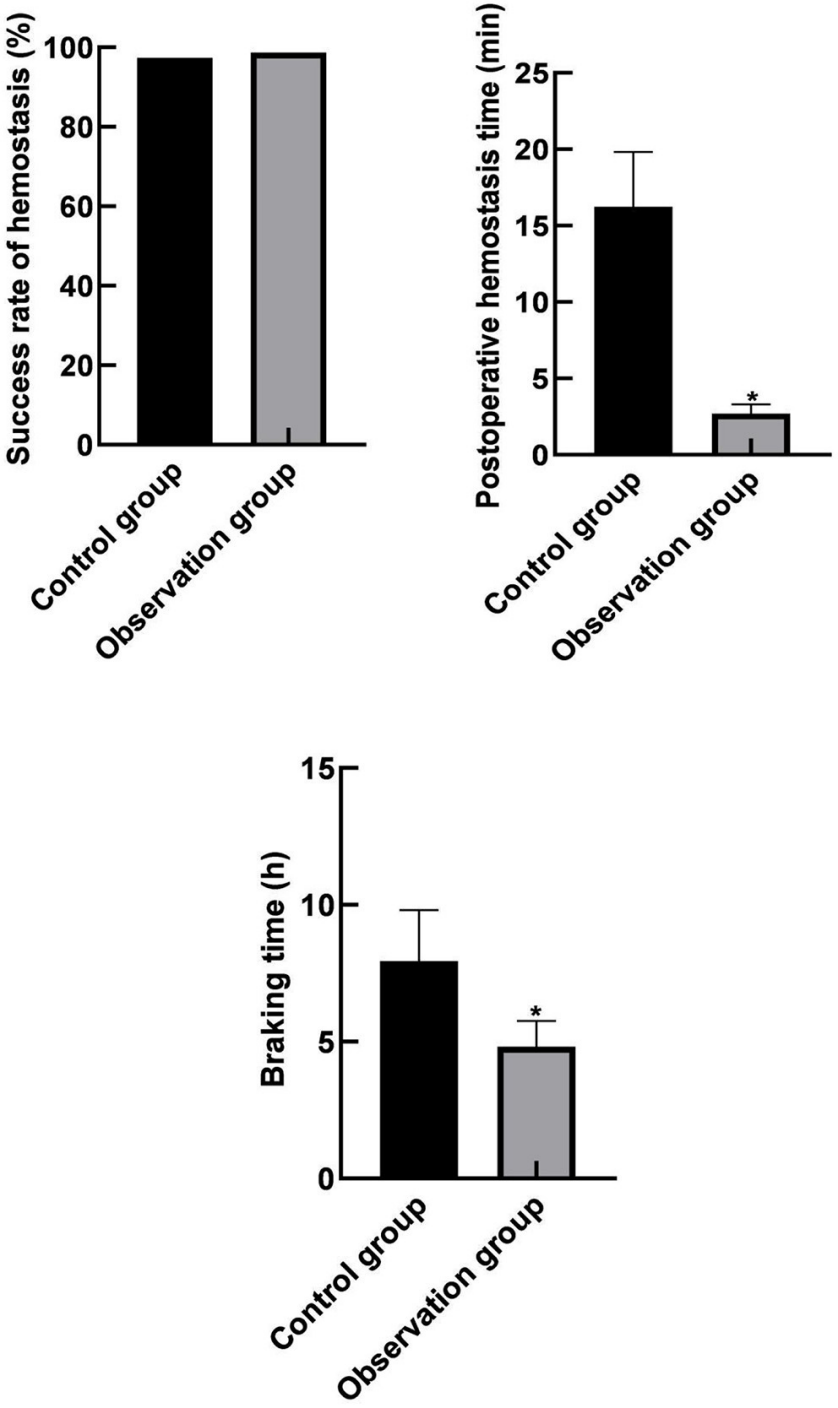
Comparison of hemostatic efficacy indicators between the two groups. Compared with the control group, *p < 0.05.

There were 147 cases of successful hemostasis in the control group, and the success rate of hemostasis was 97.35% (147/151), 149 cases of successful hemostasis in the observation group, and the success rate of hemostasis was 98.68% (149/151). There was no significant difference in the success rate of hemostasis between the two groups (*p* > 0.05). The hemostatic time and immobilization time (2.69 ± 0.62) min and (4.82 0.93) h in the observation group were lower than those in the control group (16.24 ± 3.58) min and (7.94 1.86) h (*p* < 0.05) as shown in Figure 1.

### Comparison of Vascular Parameters at the Puncture Site Between the Two Groups

**Figure 2:**
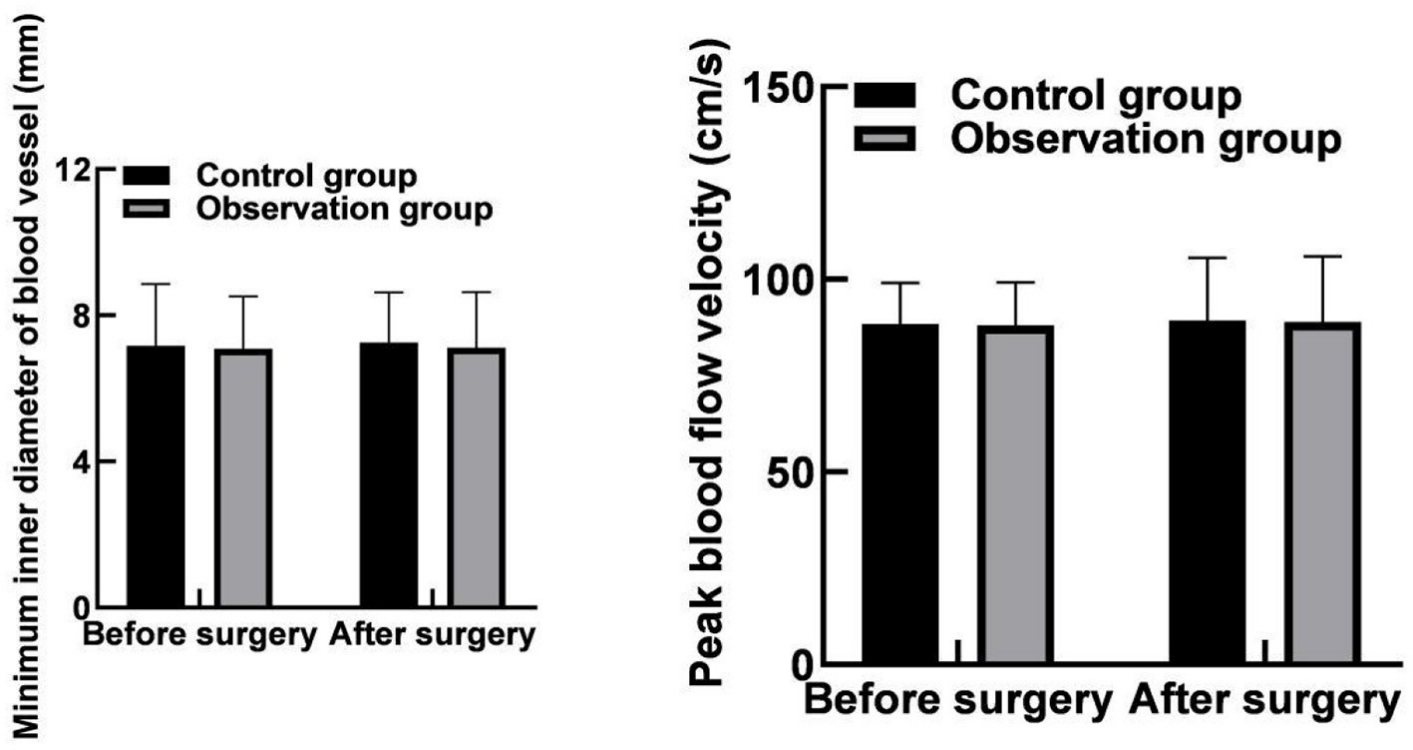
Comparison of vascular parameters at the puncture site between the two groups.

The differences in the minimum inner diameter of the puncture site and its nearby vessels and the peak velocity of blood flow between the two groups before and after the operation were not statistically significant within or between groups (*p* > 0.05) as shown in Figure 2.

### Comparison of the Comfort Level During Hospitalization Between the Two Groups

**Figure 3:**
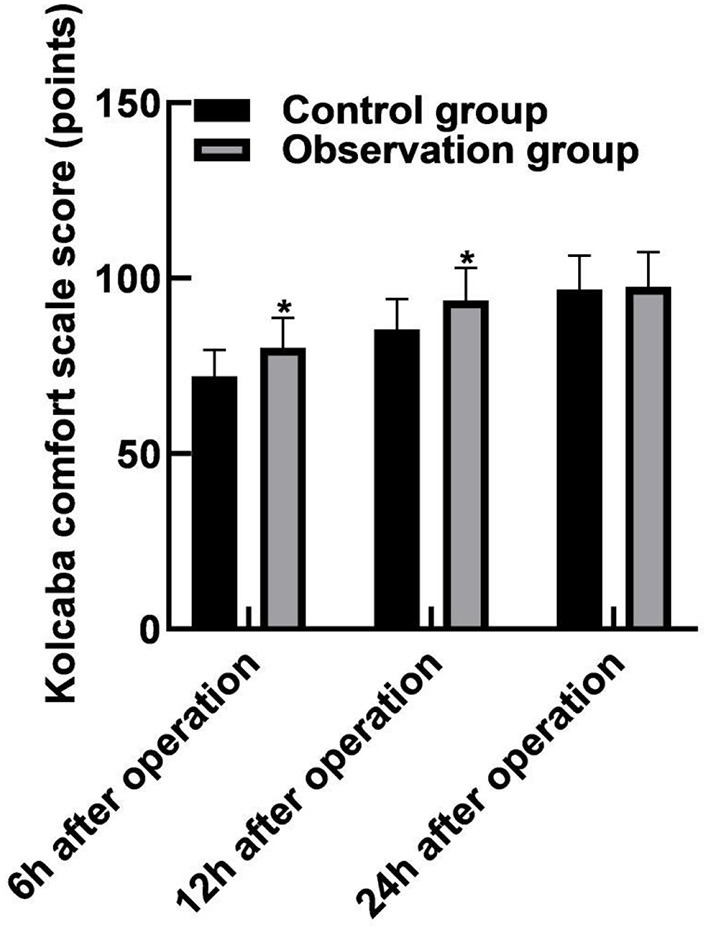
Comparison of the comfort level during hospitalization between the two groups. Compared with the control group, *p < 0.05.

The scores of the Kolcaba comfort scale in the observation group (80.16 ± 8.49), (93.65 ± 9.26) at 6 and 12 h after operation were higher than those in the control groups (72.08 ± 7.54), (85.49 ± 8.63) (*p* < 0.05). The 24-h postoperative Kolcaba comfort scale score was (97.54 ± 9.86) in the observation group and (96.82 ± 9.64) in the control group, and the difference was not statistically significant (*p* > 0.05), as shown in Figure 3.

### Comparison of Complications Between the Two Groups

**Figure 4:**
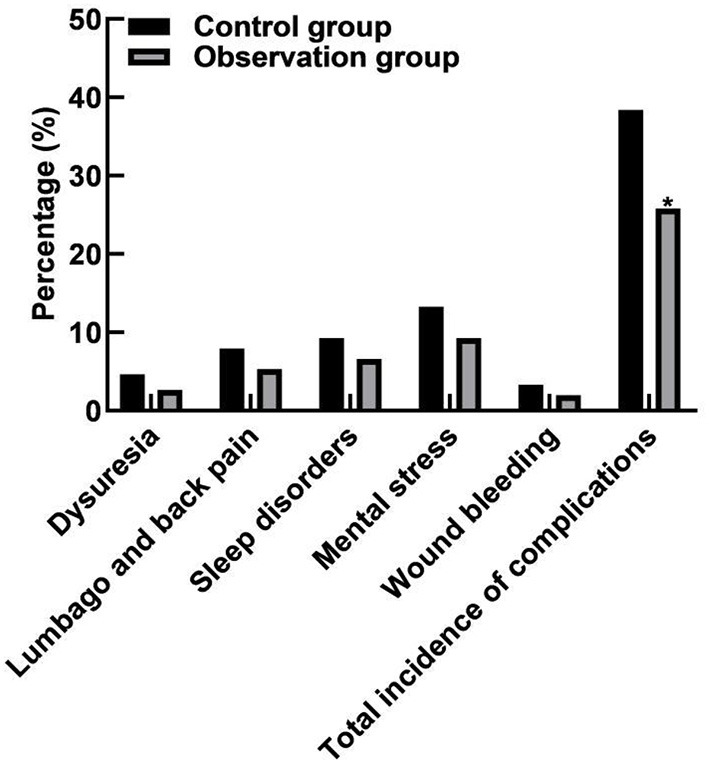
Comparison of complications between the two groups. Compared with the control group, *p < 0.05.

In the control group, there were 7 cases of dysuria, 12 cases of low back pain, 14 cases of sleep disorder, 20 cases of mental stress, and 5 cases of wound bleeding, and the total incidence of complications was 38.41% (58/151). In the observation group, there were 4 cases of dysuria, 8 cases of low back pain, 10 cases of sleep disorder, 14 cases of mental stress, and 3 cases of wound bleeding, and the total incidence of complications was 25.83% (39/151). The total incidence of complications in the observation group was lower than that in the control group (*p* < 0.05), as shown in Figure 4.

## Discussion

Cerebrovascular disease is one of the three leading causes of human death nowadays, with a high incidence, high disability, high mortality, and a high recurrence rate. Cerebrovascular interventional surgery is a commonly used method of cerebral vascular examination and treatment, it is mainly through the femoral artery or groin artery puncture into the cerebral vascular, and an interventional surgery or after check can clearly display a collateral compensatory situation of brain vascular blood flow condition and vascular anomalies, and helps the brain aneurysm, arteriovenous malformation in the diagnosis, and treatment of diseases (14–16). A small amount of antiplatelet drugs and anticoagulants is needed to maintain smooth blood vessels during the interventional surgery, but they often cause hemorrhage and hematoma at the puncture site after surgery, which can further lead to low back pain, mental stress, and dysuria. Most of the patients with cerebrovascular disease are elderly patients with poor physical function, varying degrees of impairment of limb sensation and motor ability, and abnormal coagulation function. Special attention should be paid to interventional surgery, and the postoperative puncture site is also prone to bleeding (17, 18). Therefore, how to quickly and effectively stop the bleeding is extremely important.

Traditional manual compression hemostasis, vascular stapler, and arterial compression hemostasis are currently the three commonly used hemostasis methods at the puncture site after interventional surgery. Traditional artificial compression hemostasis is simple and feasible, but compression dressing is required after hemostasis, and patients are required to stay in bed for 24 h, which can lead to many adverse reactions, such as dysuria and soreness of the waist and back caused by staying in bed for a long time (19, 20). The artery compression hemostat uses a bionic pressing plate to replace a finger, and the adhesive tape is matched with the base so that the problem with gauze that is difficult to fix in artificial compression hemostasis is solved; meanwhile, nursing staff can directly observe the hemostasis of patients and timely handle sudden unexpected situations at puncture sites. Although the arterial compression device has a good hemostatic effect, its postoperative patients need to be braked for 8 h to move freely, which easily causes complications, such as subcutaneous thrombosis and hematoma and is not conducive to the recovery of patients and affects their comfort levels (21, 22).

The results of this study showed that the hemostatic time and the braking time after operation in the observation group were lower than those in the control group. These results indicated that hemostasis with a vascular stapler could shorten the hemostasis time, and the postoperative braking time for patients was short. The reason for this was analyzed as the vascular stapler was a new type of hemostatic device that could directly suture and puncture blood vessels for rapid hemostasis; besides, the amount of blood leakage from blood vessels after suture was less, the local hematoma was less, and the hemostatic effect was better. Moreover, the results of this study showed that the minimum inner diameter and the peak velocity of blood flow at the puncture site and its nearby vessels in the two groups were not statistically different before and after the operation within and between groups. These results indicated that the use of vascular staplers for hemostasis did not increase the risk of lower extremity ischemia as compared with the arterial compression hemostats for hemostasis.

The results of this study showed that the Kolcaba comfort scale scores of the observation group at 6 and 12 h after the surgery were higher than those of the control group. It indicated that the patients using vascular stapler for hemostasis after the neural intervention had a higher comfort level. The reason was analyzed as follows: The hemostatic principle of arterial hemostat was mainly to compress the vascular puncture point with the pressure plate and fix it with adhesive tape for hemostasis, which required long-time compression of the artery, with high requirements on the strength and time of compression. Generally, the bandage could not be removed until 8 h, which might induce a variety of complications, such as lower extremity arterial ischemia, vasovagal reflex, and others, seriously affecting the patient's motor ability, and the patient's comfort level was poor. The vascular suture device directly sutures the punctured blood vessels through suture lines, which are simple to operate and rapid to stop bleeding and can significantly reduce the compression on the artery. Moreover, the patient's limb braking time is short, and the patient's motor ability is not affected, so, the patient's comfort level is high (23, 24). However, 24 h after the surgery, there was no significant difference in the scores between the two groups based on Kolcaba Comfort Scale. It indicated that the comfort levels of the patients between the two groups were not significantly different 24 h after the operation. The reasons were analyzed as follows: The patients using the arterial compression hemostat for hemostasis could move slightly on the bed 8 h after the operation so that the patients could relax and do not feel nervous all the time. This could improve sleep quality and significantly increase the comfort level. Moreover, the results of this study showed that the total incidence of complications in the observation group (25.83%) was lower than that in the control group (38.41%). These results indicated that hemostasis with a vascular stapler could significantly reduce the postoperative complications of patients. Compared with the arterial compression hemostat for hemostasis, although the vascular stapler has a better hemostatic effect, the applicability of the arterial compression hemostat for hemostasis is more extensive. The vascular suture device needs to suture the blood vessels directly. Therefore, the vascular suture device is not suitable for patients with a puncture point at the bifurcation of the blood vessels, a vessel diameter of <4 mm, atherosclerosis in the blood vessel wall, repeated fitting of the blood vessel wall, and severe tortuous iliac artery.

## Conclusion

For patients with ischemic cerebrovascular disease undergoing femoral artery puncture intervention, the use of vascular closure devices can stop bleeding quickly, which can significantly shorten the bleeding time, and the postoperative braking time of patients is short, with high comfort and fewer complications.

## Data Availability Statement

The original contributions presented in the study are included in the article/supplementary material, further inquiries can be directed to the corresponding author/s.

## Ethics Statement

The studies involving human participants were reviewed and approved by the Ethics Committee of Taizhou First People's Hospital. The patients/participants provided their written informed consent to participate in this study.

## Author Contributions

CX is responsible for the design and guidance of the study. YZ is responsible for the inclusion of cases, the detection of results, and the writing of the paper. Both authors of this study have made equal contributions.

## Funding

This study was supported by the Zhejiang Medical and Health Science and Technology Plan Project (No. 2020KY1041), Zhejiang Public Welfare Technology Application, and the Social Development Project (No. LGF19H090001).

## Conflict of Interest

The authors declare that the research was conducted in the absence of any commercial or financial relationships that could be construed as a potential conflict of interest.

## Publisher's Note

All claims expressed in this article are solely those of the authors and do not necessarily represent those of their affiliated organizations, or those of the publisher, the editors and the reviewers. Any product that may be evaluated in this article, or claim that may be made by its manufacturer, is not guaranteed or endorsed by the publisher.
